# Identification of a Novel WFS1 Mutation Using the Whole Exome Sequencing in an Iranian Pedigree with Autosomal Dominant Hearing Loss

**DOI:** 10.22038/ijorl.2021.48471.2602

**Published:** 2021-05

**Authors:** Javad Mohammadi-asl, Nader Saki, Masoud Dehdashtiyan, Mostafa Neissi, Farideh Ghanbari Mardasi

**Affiliations:** 1 *Noorgene Genetics Lab, Ahvaz, Iran.*; 2 *Hearing Research Center, Ahvaz Jundishapur University of Medical Sciences, Ahvaz, Iran.*; 3 *Department of the Pediatrics, Imam Khomeini Hospital, Ahvaz Jundishapur University of Medical Sciences, Ahvaz, Iran. *; 4 *Department of Genetics, Khuzestan Science and Research Branch, Islamic Azad University, Ahvaz, Iran.* * Department of Genetics, Ahvaz Branch, Islamic Azad University, Ahvaz, Iran.*; 5 *Department of Medical Genetics, Faculty of Medicine, Tehran University of Medical Sciences, Tehran, Iran.*

**Keywords:** Hearing Loss, Novel Mutation, Next Generation Sequencing (NGS), Whole Exome Sequencing (WES), WFS1

## Abstract

**Introduction:**

Sensorineural hearing loss is the most frequent type of hearing impairment in the human population. Genetic factors account for over 60% of hearing loss in patients. This is a genetically heterogeneous sensorineural disorder.

**Case Report:**

We carried out whole exome sequencing (WES) to screen hearing loss candidate genes in a member of an Iranian family with hearing loss. The Sanger process was used to sequencing the variant in the family members. A novel mutation (c. 559C > T) was found in the WFS1 gene (in exon 5) in the patient leading to a heterozygous missense mutation (p.L187F). Furthermore, it co-segregated with HL in the family. All affected individuals in the family had severe-to-profound HL.

**Conclusion:**

This survey is the first to describe WFS1 related HL in the Iranian population. Our data propose that the WFS1-p.L187F mutation is the pathogenic variant for autosomal dominant nonsyndromic hearing loss. Our results extend the range of the WFS1 gene mutations.

## Introduction

Hearing loss (HL) is the most frequent sensory disorder in people and occurs approximately in 1.86 per 1000 live *births* (1). Recent studies *have* shown that genetic and *environmental factors* have been implicated in the *pathogenic mechanisms underlying* HL. Hereditary HL accounts for almost 68% of all congenital hearing loss patients and 54% of HL at *four years* of *age* (2-3).

Moreover, 70% of genetic HL patients demonstrate nonsyndromic HL that are usually not *correlated* with other *abnormalities.* The *inheritance* *mode* of nonsyndromic hearing loss is *classified in* autosomal dominant (AD), autosomal recessive (AR), mitochondrial, and X-linked manner. It is expected that 80% of genetic patterns of nonsyndromic HL are AR (ARNSHL) and the remaining 20% are AD (ADNSHL) (3). 

So far, more than 130 loci and over 70 genes have been recognized in terms of NSHL (http://hereditaryhearingloss.org); therefore, this is genetically heterogeneous. With the appearance of the whole exome sequencing (WES) based on next-generation sequencing (NGS), heterogeneous medical conditions have become accessible for the discovery of the causative genes and diagnosis (4).

In the present study, we examined an Iranian family with targeted NGS and recognized a novel mutation in the WFS1 gene (c. 559C > T) which is most likely correlated with nonsyndromic HL. This represents the first study of a mutation on the WFS1 gene that causes NSHL in the Iranian population as this mutation was not observed in any of the mutation databases.

## Case Report

**Fig 1:**
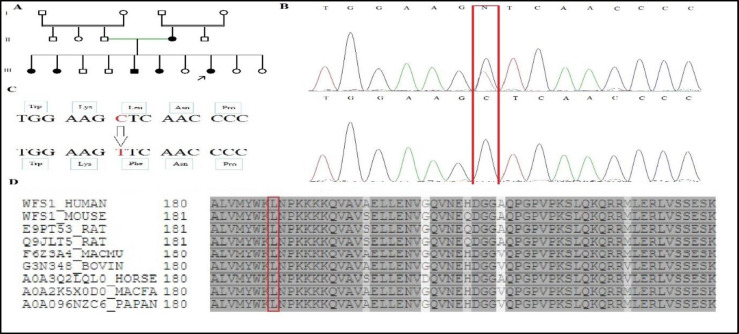
(A) Pedigree of a family with the p.L187F mutation. Filled symbols indicating affected individuals. (B) Partial DNA sequences of the *WFS1 *gene showing the c.559C>T change in the family members and a normal control. (C) The amino acid changes are caused by the changes in the DNA sequence. (D) Multiple alignments of the *WFS1* in different species. The amino acid sequence of human *WFS1 *is aligned with sequences of other species. The =arrow marks the p.L187F mutation

This study was conducted in an Iranian family presenting as autosomal dominant NSHL. A written informed consent form was collected before participant inclusion in the survey based on the guidelines of the Ethics Committee of Iran’s Ministry of Health and Medical Education. The proband, a 12-year-old female, (Figure 1A) showed a dominantly NSHL, which was severe-to-profound symmetrical bilateral HL. There were no other abnormal physical findings during the examination, including vestibular function tests and electrocochleogram tests. *Magnetic resonance imaging *revealed a normal internal auditory canal. Moreover, her siblings (III-1; 26 years old, III-2; 24 years old, III-5; 18 years old, and III-6; 16 years old) were diagnosed with *hearingloss*.

A comprehensive assessment of the health history of the family did not recognize any other *features* of *syndromic* disorders.

Genomic DNA was obtained from *human blood* leukocytes samples using salting-out methods. Qualified genomic DNA from the proband (III-8) of this family was employed for sequencing of candidate genes using the WES technique by Macrogen, Seoul, South Korea. For analysis and *interpretation* of the sequencing data, the *public* mutation and polymorphism databases, such as 1000 Genomes Project and EXAc Browser were used in this study. Only the low-*frequency variants* (below 1%) were selected. Following that, to verify the true positive of the novel mutation detected by WES, direct Sanger sequencing was performed in the patient and family members.

A novel heterozygous missense mutation c. 559C > T (CTC > TTC) was detected in the WFS1 gene that was subsequently co-segregated with the disease in the family (Figure 1B). This variant has not been described in public databases, such as 1000 Genome and ExAC. Based on the ACMG guidelines, the c. 559 C>T variant categorized as a variant of uncertain significance. The findings showed that each patient was heterozygous for c. 559 C>T variant, and none of the healthy members of the family carried this variant that was co-segregated with *deafness.*The missense mutation identified in exon 5 of the WFS1 gene (c. 559 C>T) (Figure 1C) led to a leucine to phenylalanine substitution (Leu187Phe). This missense mutation was located in the cytoplasmic region of the wolframin protein, which was predicted to be pathologic by prediction programs. The proband in the family, patient with WFS1 gene mutation c. 559C>T, along with his affected sisters and brother, suffered from prelingual ADNSHL at birth. Audiological assessment of the proband showed severe to profound HL. The WFS1 sequence alignment from eight diverse species (Mouse, Homo sapiens, Macmu, Rattus, Bovin, Horse, Papan and Macfa) is given in Figure 1 D. This finding revealed that the leucine amino acid was a highly *conserved *residue in the *evolution* of wolframin protein, demonstrating that this mutated amino acid is essential for appropriate protein function. Furthermore, the WFS1c. 559 C>T, p.L187 F Missense mutation was recognized in her affected mother and siblings in the heterozygous state with the dominant inheritance pattern.

## Discussion

More than 130 loci and over 70 genes have so far been associated with hereditary auditory disorder [http://hereditaryhearingloss. Org], the majority of which was recognized throughout using the *typical* linkage analysis. A *high *frequency of sensorineural hearing loss remains genetically unclear. However, the WES technique presents a potent and inexpensive modality to recognize disorder correlated mutations for Mendelian disorders (5-8).

In the present study on a family with deafness, the WES initially showed a novel mutation in the WFS1 gene to be the underlying cause of the impairment in the family. Subsequently, direct Sanger sequencing proved the existence of this mutation. All these tests recommend that these patients with HL in the present pedigree carried the heterozygous C729T mutation, which demonstrated co-segregated with HL. This mutation was not present in the healthy *people*in the family with normal hearing. The WFS1 gene (NM-006005.3), which is located at 4p16.1, spans approximately 33.4 kb of DNA, and is transcribed into a cDNA of 3.6 kb. This gene encodes wolframin ER transmembrane glycoprotein of 890 amino acids that belongs to the *transmembrane protein* family (9). The predictions of secondary structure recognized three domains in this family, including a hydrophilic domain at the N-terminal, a central *hydrophobic domain consisting* membrane-spanning subunits, and a hydrophilic carboxy tail at the C-terminal (9,10). 

No important homologies were detected in the databases; therefore, this defines a novel transmembrane protein family. So far, the role of wolframin is entirely unidentified (10). Wolframin is considered to play a function in the regulation of cellular Ca (2+) homeostasis by modulating the *free endoplasmic reticulum Ca*^2+^ *concentration* (11). 

This missense mutation p.L187F recognized in the family caused a substitution of the non-polar aliphatic Leu187 by the non-polar aromatic Phe, which is situated in the cytoplasmic region of the wolframin protein. We believe this p.L187F mutation to be pathogenic since the mutated leucine amino acid is evolutionarily conserved (Figure 1D). In addition, bioinformatics software, such as SIFT, Mutation Taster, and polyphen predicted that this mutation would be damaging and disease-causing. Therefore, high conservation of the leucine amino acid at position 187 of the WFS1 protein presents confirmation that this amino acid is essential for suitable protein function.

## Conclusion

In conclusion, WES has been verified to be a powerful technique to recognize causative rare variants. The use of this technique in the present study resulted in the recognition of a novel variant in the WFS1 gene in a family of Iranian origin, which causeed NSHL.
